# Social diversity and access to healthcare in Europe: how does European Union’s legislation prevent from discrimination in healthcare?

**DOI:** 10.1186/s12889-020-09494-8

**Published:** 2020-09-14

**Authors:** Marcin Orzechowski, Marianne Nowak, Katarzyna Bielińska, Anna Chowaniec, Robert Doričić, Mojca Ramšak, Paweł Łuków, Amir Muzur, Zvonka Zupanič-Slavec, Florian Steger

**Affiliations:** 1 Institute of the History, Philosophy and Ethics of Medicine, Ulm University, Parkstraße 11, 89073 Ulm, Germany; 2 Center for Bioethics and Biolaw, Faculty of Philosophy, University of Warsaw, Warsaw, Poland; 3 Department of Social Sciences and Medical Humanities, Faculty of Medicine, University of Rijeka, Rijeka, Croatia; 4 Institute for History of Medicine, Faculty of Medicine, University of Ljubljana, Ljubljana, Slovenia; 5 Department of Public Health, Faculty of Health Studies, University of Rijeka, Rijeka, Croatia

**Keywords:** Healthcare inequality, European Union, Public nondiscriminatory policy, Minority group, Ethnicity, Religious belief, Sexual orientation

## Abstract

**Background:**

Social diversity can affect healthcare outcomes in situations when access to healthcare is limited for specific groups. Although the principle of equality is one of the central topics on the agenda of the European Union (EU), its scope in the field of healthcare, however, is relatively unexplored. The aim of this study is to identify and systematically analyze primary and secondary legislation of the EU Institutions that concern the issue of access to healthcare for various minority groups. In our research, we have concentrated on three features of diversity: a) gender identity and sexual orientation, b) race and ethnicity, and c) religion or belief.

**Method and materials:**

For the purpose of this analysis, we conducted a search of database Eur-Lex, the official website of European Union law and other public documents of the European Union, based on specific keywords accompanied by review of secondary literature. Relevant documents were examined with regard to the research topic. Our search covered documents that were in force between 13 December 2007 and 31 July 2019.

**Results:**

Generally, the EU legal system prohibits discrimination on grounds of religion or belief, racial or ethnic origin, sex, and sexual orientation. However, with regard to the issue of non-discrimination in access to healthcare EU secondary law provides protection against discrimination only on the grounds of racial or ethnic origin and sex. The issue of discrimination in healthcare on the grounds of religion or belief, gender identity and sexual orientation is not specifically addressed under EU secondary law.

**Discussion:**

The absence of regulations regarding non-discrimination in the EU secondary law in the area of healthcare may result from the division of competences between the European Union and the Member States. Reluctance of the Member States to adopt comprehensive antidiscrimination regulations leads to a situation, in which protection in access to healthcare primarily depends on national regulations.

**Conclusions:**

Our study shows that EU antidiscriminatory law with regard to access to healthcare is fragmentary. Prohibition of discrimination of the level of European binding law does not fully encompass all aspects of social diversity.

## Background

Non-discriminatory access to healthcare services is one of the basic principles of health law and of medical ethics [1]. As an aspect of the right to health, it constitutes a human right and is anchored in various international treaties [2], e.g. the preamble of the Constitution of the World Health Organization of 1948, the International Covenant on Economic, Social and Cultural Rights of 1966 or the International Convention on the Protection of the Rights of All Migrant Workers and Members of their Families of 1990. With regard to medical ethics, it touches the essential principle of justice in medical care [3]. In this context, legal and organizational structures should guarantee equity in access to healthcare for all, including members of minority groups. Without such guarantees, societies run the risk of progressing alienation of these groups and put in question democratic principles of social order in question.

For several years now, social diversity has been addressed by experts as an important issue for healthcare [4, 5]. Literature on the topic highlights various characteristics of diverse social groups such as the place of residence, race or ethnicity, occupation, gender, religion, education, socio-economic status, and social capital [6] as well as language, nationality, sex, gender identity, sexual orientation, geographical origin, disability, and age [7]. When access to healthcare is limited for specific social groups, diversity of a society can have a profound impact on the provision of medical care and healthcare outcomes [8–10]. Therefore, in this context, access to healthcare for members of diverse social groups is crucial.

Equity of access to healthcare is a major social determinant of health and can be seen as a strategy for addressing health inequalities. Equal access to healthcare is not limited to physical restrictions of entry but it is central to equality of medical care. It can be defined as provision of medical treatment that does not vary in scope or quality because of groups’ characteristics or particular aspects of individuals composing these groups. Health inequities or healthcare discrimination are systematic differences in health that are unjust and could be avoided by reasonable means [11].

Access to healthcare may be hindered by numerous barriers [2]. Research shows that inequalities in access and discrimination of minorities in healthcare are common and tend to affect members of vulnerable groups, e.g. migrants, religious minorities, persons with complex medical needs, e.g. patients with chronic conditions such as cancer or diabetes or requiring surgical interventions [12, 13]. Discrimination in healthcare can be based on several characteristics, such as income, education, occupation, gender, race, ethnicity or religion and is a well-documented challenge to equal access in healthcare [14–16]. For example, patients with lower socioeconomic status consistently report more discrimination compared to patients with high socioeconomic status. According to Arpey et al., physicians are less likely to perceive patients with lower income as intelligent or independent. This has an impact on clinical decisions such as delaying diagnostic testing or avoidance of referral to specialty care [17].

Hanssens et al. argue that discrimination in healthcare has a double negative effect [18]. First, it prevents equal access and thus the fair chance of getting needed healthcare. Second, it leads to diminished trust and confidence in healthcare professionals and healthcare system. Both these outcomes can have cumulative negative effects for various disadvantaged groups of population, thus contributing to health inequalities. They may lead to lack of attention to their healthcare needs, worse health outcomes, increased costs of medical care, and progressing social marginalization [19].

Vulnerable groups vary with regard to several characteristics, i.e. sexual orientation, gender, ethnicity, race or religion. Non-heteronormative persons, such as lesbians, gays, bisexuals, and transgender persons, experience difficulties in access to the healthcare system as a result of prejudicial and discriminatory behavior and social stigmatization [20–23]. This can directly contribute to poorer health status of the members of sexual minorities [24]. Gender inequality, as a systemic issue, may contribute to unequal access to healthcare resources, and as such contributes to unequal health outcomes [25]. However, studies suggest that healthcare organizations are mostly gendered, which means that male and female patients are treated differently [26]. Within such arrangements, non-binary persons are often left out.

As a result of migration flows in recent years, an increasing religious, ethnic, and cultural diversity of European societies is observable. In the context of access to healthcare, members of these minority groups experience barriers that include restricted legal entitlements to health services for certain groups of migrants such as asylum seekers or undocumented migrants [27], e.g. provision of free specialist medical care; administrative obstacles [28], e.g. access to ambulatory care only with a valid healthcare voucher provided by welfare centers in Germany; language barriers [29] or the presence of xenophobia and racism among healthcare professionals [30, 31].

In our research we concentrate on investigation of structural inequities in access to healthcare. Discrimination in this area can function on multiple levels, ranging from individual to structural [32]. While in case of individual discrimination exclusion operates on the level of particular individual encounters, structural inequalities entail operation of rules and procedures that disadvantage particular minority groups. In such a case, the state or the agency establish through norms, rules, regulations or procedures either a permissive or a prohibitive context. Legal act can enforce, enable or condone inequalities, or can outlaw it and seek to redress its effects [33]. Through this, they can influence individual behavior and may motivate the exclusion or inclusion of persons that do not necessarily follow the dominant social norms.

Social diversity is one of the fundamental features of the European Union (EU) – it constitutes one of its main values but it is also a regulatory challenge in several areas. Although the right of access to healthcare and the right to benefit from medical treatment is one of the central topics on the agenda of the EU [34], the EU regulations relating to discrimination in access to healthcare remain relatively unexplored in the topic literature [35, 36]. Therefore, we decided to analyze systematically the regulations, guidelines, and documents of the EU institutions that concern the question of access to healthcare for minority groups. The main aim of the research is presentation of a regulatory framework on this issue. As the legislative approach to diversity is under constant development in response to new challenges of diversity, our goal is to present the approach of the EU institutions to the issue of diversity and equal access to healthcare. Thus, the key research question is: how are the issues of social diversity and access to healthcare regulated in the documents of the EU institutions? In our attempt to answer this question, we have focused on the three following dimensions of diversity – a) gender identity and sexual orientation, b) race and ethnicity, and c) religion and belief – and examined how the EU regulations target the issue of access to healthcare of persons with these characteristics.

## Methods

### Method

The search for relevant documents was conducted in a two-step procedure. First, we conducted a search of the database Eur-Lex, which is the repository of legal documents of the European Union. The goal of the search was to find answers to the research question and related empirical evidence. Several keywords were combined in order to identify documents relevant for the analysis. In the search we have combined the main keywords “health” and “healthcare” with the following keywords: “race”, “ethnicity”, “ethnic minorities”, “religion”, “religious beliefs”, “denomination”, “gender”, “transsexual”, “homosexual”, “women”, “sexual orientation”. We searched for these keywords in titles as well as in the texts of the documents.

Our search covered documents that were in force between 13 December 2007 and 31 July 2019. The date 13 December 2007 was chosen as the date of signing of the Lisbon Treaty that regulates the distribution of competences in various policy areas, among others healthcare, between the EU and the Member States.

Second, the search of regulatory documents was accompanied by review of secondary literature in order to identify possible documents missed in the first phase and to provide a comprehensive view, understanding, and context of the topic. This study is a critical analysis of the regulations on equal access to healthcare. Because our goal is to investigate how and to what extent the EU regulations take diversity into account, a survey of regulatory documents alone is insufficient, but requires comprehensive interpretation and understanding, also in relation to the contexts for which they were designed. This approach is also justified by the fact that the regulatory documents do not rely on a common and fixed vocabulary in this matter. The vocabulary has expanded and evolved over the years and has not been codified, e.g. the evolution of the concepts of sex or gender.

The study was designed as documentary research with legal documents as source materials. The documents serve as evidence of policy approach adopted by the EU. As official legislative texts published by the European Union, they meet all of the four criteria according to Scott and Marshall, which are authenticity, credibility, representativeness, and meaning [37]. Documents with relevant titles were examined to identify provisions pertaining to the research topic. Excluded were documents that do not relate to the three features of diversity that were the object of the research as well as documents that do not refer to the issue of access to healthcare, e.g. anti-discrimination laws in healthcare as a workplace. Excluded were also documents that did not have the status of official documents, such as proposals that were not accepted by the official organs of the EU, e.g. Equal Treatment Directive of 2008. Through this procedure, we have selected 15 documents, which are included in this analysis.

Documents identified in both phases were searched using keywords to determine paragraphs relevant to the research topic. The content of the documents was analyzed manually and systematically categorized, with regard to the following items: issuing institution, legal validity, area of protection, included legal measures. The relevant documents were independently analyzed by at least two researchers. Drawing on the key findings from the analysis, we developed a narrative synthesis.

### Materials

The EU law can be divided into two main types: primary and secondary legislation. Primary legislation encompasses treaties, which represent the constitutional framework of the EU. Secondary legislation consists of laws made by the EU institutions. It encompasses binding instruments, i.e. regulations, directives, decisions, and non-binding instruments, i.e. recommendations, opinions, resolutions, and declarations. EU secondary legislation is initiated by the European Commission and has to be approved by both the European Parliament as well as the European Council. Regulations are directly applicable in all Member States. Directives apply to Member States and are binding in relation to the issue addressed in them. Directives need to be incorporated into national law but each Member State can choose their own appropriate measures to do so [38, 39]. Recommendations, opinions, resolutions, and declarations provide views and statements of the EU institutions on a particular issue, outline joint goals and intentions in relation to a possible future direction or common actions of the European Union. Programmes of community action are documents issued by the EU Commission, in which it broadly states the actions and legislation it intends to pursue in a five-year framework. On the basis of an action programme, the Commission proposes policy initiatives and legislative initiatives. Although these measures – so-called soft law – lack legally binding effect, they constitute guidelines that can have an impact on policy development, influence national regulations of the Member States and provide information on policy aims in a particular area [40]. The materials analyzed below encompass primary and secondary legislation of the European Union, including both binding and non-binding instruments.

## Results

We have identified 15 binding and non-binding EU documents referring to the research question (see Table 1).

**Table 1:** Outline of the EU documents included in the analysis

LEGISLATION	SOURCE	LEGAL VALIDITY
**Primary legislation**		
The Treaty on European Union	Official Journal of the European Union C236. 26.10.2012 [41]	binding
The Treaty on the Functioning of the European Union	Official Journal of the European Union C236. 26.10.2012 [42]	binding
Charter of Fundamental Rights of the European Union	Official Journal of the European Communities C364. 18.12.2000 [43]	binding
**Secondary legislation**		
Council Directive 2000/43/EC of 29 June 2000 implementing the principle of equal treatment between persons irrespective of racial or ethnic origin	Official Journal of the European Union L180. 19.7.2000 [44]	binding
Council Directive 2004/113/EC of 13 December 2004 implementing the principle of equal treatment between men and women in the access to and supply of goods and services	Official Journal of the European Union L373. 21.12.2004 [45]	binding
Directive 2011/24/EU of the European Parliament and of the Council of 9 March 2011 on the application of patients’ rights in cross-border healthcare	Official Journal of the European Union L88. 4.4.2011 [46]	binding
Council Conclusions on Common values and principles in European Union Health Systems	Official Journal of European Union C146. 22.6.2006 [47]	non-bindng
White Paper Together for Health: A Strategic Approach for the EU 2008–2013	COM(2007) 630 final. 23.10.2007 [48]	non-binding
Decision No 1786/2002/EC of the European Parliament and of the Council of 23 September 2002 adopting a programme of Community action in the field of public health (2003–2008)	Official Journal of the European Communities L271. 9.10.2002 [49]	binding
Decision No 1350/2007/EC of the European Parliament and of the Council of 23 October 2007 establishing a second programme of Community action in the field of health (2008–13)	Official Journal of the European Union L301. 20.11.2007 [50]	binding
Regulation (EU) No 282/2014 of the European Parliament and of the Council of 11 March 2014 on the establishment of a third Programme for the Union’s action in the field of health (2014–2020) and repealing Decision No 1350/2007/EC	Official Journal of the European Union L86. 21.3.2014 [51]	binding
Council Recommendation of 9 December 2013 on effective Roma integration measures in the Member States	Official Journal of the European Union C378. 24.12.2013 [52]	non-binding
**Preparatory acts**		
Communication from the Commission to the European Parliament, the Council, the European Economic and Social Committee and the Committee of the Regions: Renewed social agenda: Opportunities, access and solidarity in twenty-first century Europe	COM(2008) 412 final. 2.7.2008 [53]	non-binding
Communication from the Commission to the European Parliament, the Council, the European Economic and Social Committee and the Committee of the Regions: Solidarity in Health: Reducing Health Inequalities in the EU	COM(2009) 567 final. 20.10.2009 [54]	non-binding
Communication from the Commission to the European Parliament, the Council, the European Economic and Social Committee and the Committee of the Regions: An EU Framework for National Roma Integration Strategies up to 2020	COM(2011) 173 final. 5.4.2011 [55]	non-binding

Although the principle of equality is clearly stated among the goals of the European Union provided by the EU primary law, the treaties of the European Union do not directly address the issue of equal access to healthcare for members of minority groups. Generally, the principles of the EU set down in the treaties include respect for the rights of people belonging to minorities. Article 2 of the Treaty on European Union formulates it as following: “The Union is founded on the values of respect for human dignity, freedom, democracy, equality, the rule of law and respect for human rights, including the rights of persons belonging to minorities” [41]. On this basis, the EU should combat social exclusion and discrimination and shall promote social justice and solidarity [41–43]. With regard to this, the Charter of Fundamental Rights (CFR) of the EU stipulates in Article 35 that: “Everyone has the right of access to preventive health care and the right to benefit from medical treatment under the conditions established by national laws and practices” [43]. The Charter maintains in the Article 21 the right to non-discrimination on any ground, such as sex, race, ethnic or social origin, religion or belief, sexual orientation or membership in a national minority. However, explicit policy measures to achieve compliance with the principle of equality in access to healthcare are not specifically mentioned in the primary-law instruments.

Within the applicable EU regulatory framework, two secondary-law instruments assume an essential role in guaranteeing equal access to medical services for minority groups: Directive 2000/43/EC [44] and Directive 2004/113/EC [45]. Both legal instruments stipulate that they apply to all persons, in the public and private sectors in relation to healthcare. However, both directives cover only limited areas. In the case of the Directive 2000/43/EC, it is protection against discrimination in healthcare on grounds of racial or ethnic origin [44]. The Directive 2004/113/EC guarantees protection against sex discrimination in access to, among others, healthcare services [45]. The principle of equal treatment in both directives prohibits direct or indirect discrimination based on ethnicity, race or sex. In this context, discrimination means less favorable treatment as well as apparently neutral provisions or practices that would put particular persons at a disadvantage as compared to other persons. Both directives aim to ensure a common level of protection across the European Union but they also allow the Member States to provide a higher level of protection through national regulations, for example through Art. 6 paragraph 1 of the Directive 2000/43/EC: “Member States may introduce or maintain provisions which are more favourable to the protection of the principle of equal treatment than those laid down in this Directive” [44].

In addition to these two central legal acts, the Directive 2011/24/EU on the application of patients’ rights in cross-border healthcare acknowledges that the health systems constitute a central component of social protection within the EU and contribute to social cohesion and justice [46]. However, the Article 4 point 3 specifically refers to the principle of non-discrimination only with regard to nationality of patients from other Member States: “The principle of non-discrimination with regard to nationality shall be applied to patients from other Member States” [46]. The Directive recognizes the existence of common rules and principles shared across the EU, such as equity and solidarity, and stresses that healthcare systems should respond to the needs of the population and patients.

The three-abovementioned directives directly regulate antidiscrimination in access to healthcare in the EU. Additionally, the European Institutions address the issue in a number of further measures and guidelines that highlight the importance of equal provision of medical services.

Substantial in this context is a resolution of the European Council from 2006, which stresses the principal values of the Community in relation to access to healthcare: universality, good quality care, solidarity, and equity in access to healthcare, which should be provided according to the need, regardless of ethnicity, gender, age or social status [47]. However, other characteristics of minority groups, such as sexual orientation or religion, are not mentioned in this resolution. This resolution constituted a basis for a number of following initiatives that focused on the issue of discrimination in healthcare.

The European Commission plays here a central role. In a number of documents, the Commission stresses the influence of factors such as discrimination, stigmatization, and barriers in accessing healthcare, which result in poor health levels for members of the vulnerable and socially excluded groups, including people from migrant or ethnic minority backgrounds [48, 53, 54]. In this respect, the aim of the EU should be to tackle inequalities in health on grounds of ethnicity, gender, age, and social status. Once again, characteristics such as religion or sexual orientation are not explicitly mentioned in these guidelines. In order to achieve the assumed aim, the European Commission aims to launch appropriate EU-level actions that should encompass initiatives in collaboration with the Member States in promotion and improvement of access to healthcare, development of appropriate health services, health promotion and preventive care for migrants and ethnic minorities, as well as identification and exchange of good practices in the healthcare sector [54]. Within its area of responsibility, the European Commission should propose a comprehensive directive to combat discrimination in healthcare. In its scope, it should complement Directives 2000/43/EC and 2004/113/EC and cover the characteristics not included in previous legislations, such as religion or belief and sexual orientation [53].

Focus on promotion of equity and solidarity in healthcare is visible in three Programmes of Community action in the field of public health for the years 2003–2008, 2008–2013, and 2014–2020 [49–51]. The overall goal of the Programmes is to attain a high level of physical and mental health and greater equality in health matters throughout the Community. To this aim, actions reducing health inequalities should be undertaken. However, these Programmes do not identify any specific actions towards this aim. Moreover, the focus of the Programmes does not include specific areas of inequality beyond inequalities related to gender differences.

With regard to a particular ethnic group, special attention in European guidelines receives the situation of the Roma community. Some European documents address non-discriminatory access to medical services with a focus on the specific needs of this minority group [52, 55]. The provision of healthcare for the Roma in this respect is addressed as one of the crucial areas of integration. However, as the Council of the European Union stresses, proposed integration measures should not exclude other marginalized and disadvantaged groups and should be based on the same principles in comparable conditions [52]. The specific European goal in this area is to reduce the gap in health status between the Roma and the rest of the population through provision of access to quality healthcare. Applied measures should include removal of barriers to access to the healthcare system, improving access to medical examination, prenatal and postnatal care and family planning as well as sexual and reproductive healthcare [55].

Notwithstanding the common European aims provided by these guidelines, the European Institutions acknowledge the primary role of the Member States in the area of healthcare, as stipulated by the article 168 of the Treaty on the Functioning of the European Union (TFEU) [42]. However, the European Institutions stress that this area remains within the national protection systems of the Member States, and the European Union should play a supporting role, in order to coordinate or supplement actions of the Member States, through proposals for common European policies that address the factors contributing to health inequalities [47, 54]. As the European Commission reiterates, the implementation mechanisms of the new strategy need the involvement of Member States through national programmes and legislation [48, 53–55].

## Discussion

The results of our research show that although the principle of equality is one of the basic values enshrined in treaties of the EU, binding normative acts of the European Union only marginally regulate the issue of equal access to healthcare. Directive 2000/43/EC and Directive 2004/113/EC guarantee protection against discrimination on grounds of racial or ethnic origin and sex. In addition, Directive 2011/24/EU provides protection against discrimination on grounds of nationality; however, only with regard to nationality of patients originating from other Member States. Therefore, prohibition of discrimination through legally binding acts is limited to discrimination based on racial, ethnic or gender characteristics and nationality and disregards further characteristics such as religion or sexual orientation. Because of this, the EU antidiscriminatory law is sometimes characterized as fragmentary, incoherent, and ultimately ineffective [35].

The reason for such a situation might be the division of the competences between the European Union and the Member States written down in the European Treaties. Accordingly, community action in the field of public health needs to respect the responsibilities of the Member States for the organization and delivery of health services and medical care [40, 56]. Given the Member States’ reluctance to relinquish competence in this area, the protection of equal treatment irrespective of religion, belief or sexual orientation in access to medical services depends on the guarantees offered by particular states. This leads to a situation, in which equity of access to healthcare is not harmonized across Europe and depends on the broadness of national regulations [19, 35]. Thus, individuals may enjoy an unequal level of protection depending on their geographical location [57, 58]. This can have direct effect on medical care for minorities in some Member States. As noted by Dean, in case of ethnic and religious minorities, the application of a rights-based approach to access to, among others, medical care is currently overlooked by European welfare regimes traditionally based on citizenship rather than on universality [59]. This mostly touches migrants and refugees with an undocumented status [60]. In case of missing national regulation, persisting discrimination in the form of lack of consideration for cultural practices of specific groups, violation of informed consent or professional bias could lead to adverse healthcare outcomes.

Moreover, European regulatory framework does not recognize the notion of multiple discrimination [35, 61]. This concept describes a situation, in which an individual is discriminated on the basis of several characteristics, e.g. ethnicity and sexual orientation. As it has been presented by Crenshaw in her works on limitations in black women’s access to the American labor market or with regard to domestic violence, social groups at the intersection of two or more identity categories are often overlooked by policy framework and they can become marginalized as a group to a greater degree than communities formed around one identity category [62, 63]. As sexual orientation with regard to healthcare is not regulated as a protected characteristic on the EU level, a combined claim on the basis of for example ethnicity and sexual orientation is not possible. Multiple discrimination with regard to healthcare is only regulated on the national level of some Member States [64]. Recognition of multiple discrimination in healthcare as a legal principle in European Union’s regulations would on the one hand acknowledge the complexity of various group characteristics. On the other hand, it would widen the level of protection and allow legal action against discrimination in a situation, in which a single ground of discrimination is insufficient.

Our research shows that the European Institutions continue nevertheless to promote the issue of equality in access to healthcare throughout the European Community. For this purpose, they use a variety of instruments, primarily relying on the so-called soft law measures. These practices have a normative dimension but operate beyond legally binding regulation [40]. They form policy objectives that go beyond the currently formulated regulatory framework, for example through highlighting the principle of equality in Programmes for the Union’s action in the field of health or through concentrating on particular minorities, such as the Roma community [65]. Moreover, the European Commission reiterated its ambition to further deepen European integration in this area by stating that respecting national responsibility for health systems does not mean absence of initiatives on the Community level. An initiative of that kind was the proposal for the Equal Treatment Directive of 2008 [66]. According to it, the principle of equal treatment of persons irrespective of religion or belief, disability, age or sexual orientation was to be implemented beyond the area of the labor market, thus including healthcare. The Commission’s proposal has provoked a considerable debate both within the European Institutions and between the Commission and the Member States. Because of the strong opposition of some Member States, the proposition is dormant for now [67, 68]. In order to create a more coherent, harmonious, and effective normative anti-discrimination framework in access to healthcare on the European level, there is a dire need for such comprehensive regulation. However, such anti-discrimination legislation is only a stepping stone towards more just healthcare systems. Discrimination is a function of regulatory framework but also individual actions, motivations, prejudices, as well as social and cultural norms. As Sen remarks in his book “The Idea of Justice”: “Justice is ultimately connected with the way people’s lives go, and not merely with the nature of institutions surrounding them” [69].

### Limitations

The findings from this research need to be considered in light of its limitations. First, as stated in the beginning of this paper, this analysis does not aim at provision of an exhaustive overview of anti-discrimination norms in the legislative framework of the EU, due to the fact that the framework is expanding and evolving in response to new challenges of diversity. Therefore, we have considered only three out of several dimensions of diversity. Although the concept of diversity comprises other aspects, such as age, disability or socio-economic status, our analysis focuses on the aspects that have been demonstrated in the recent literature to have an important impact on the issue of access to healthcare [2, 6, 15, 19, 24].

Second, the number of keywords used in the search was limited for practical reasons. In our opinion, keywords chosen for the research are sufficient for the identification of relevant documents. There are several other keywords possible, such as “language” or “men”; however, they rather only supplement the keywords used, without providing differentiating results. Review of the literature on the topic that accompanied our research confirms our findings in this point rather than challenges them [35, 36, 56, 61].

## Conclusions

Although the European Treaties emphasize equality, social inclusion, and combating discrimination as the common values upon which the European Union is based, secondary law of EU currently in force provides comprehensive protection only against discrimination in healthcare on the grounds of racial and ethnic origin, sex, and nationality. This leads to a situation, in which discrimination in healthcare on the grounds of religion, belief, gender identity or sexual orientation is not explicitly prohibited by the European law. Nevertheless, visible is initiative of the European Institutions, especially the European Commission, to put the issue on the European agenda. Advance of social integration and promotion of healthcare systems accessible for various minority groups has repeatedly been highlighted in several non-binding documents. Our study shows gaps in the legislation on the European level. The results of this research may lead to new policy initiatives towards protection of all minority groups in Europe.

## Data Availability

All data generated or analyzed during this study are included in this published article.

## Abbreviations

CFR: Charter of Fundamental Rights
EU: European Union
TFEU: Treaty on the Functioning of the European Union
